# Nephelometry as a simple tool to monitor the disassembly of polymeric nanoparticles

**DOI:** 10.1039/d5ay00659g

**Published:** 2025-08-26

**Authors:** Joshka Verduin, Niki Simaiaki, Freek Ariese, Govert W. Somsen

**Affiliations:** a Vrije Universiteit Amsterdam, Department of Chemistry and Pharmaceutical Sciences, Amsterdam Institute of Molecular and Life Sciences (AIMMS), Division of BioAnalytical Chemistry De Boelelaan 1108 1081 HZ Amsterdam The Netherlands g.w.somsen@vu.nl; b Centre of Analytical Sciences Amsterdam (CASA), Science Park 904 1098 XH Amsterdam The Netherlands; c LaserLaB Amsterdam, Department of Physics and Astronomy, Vrije Universiteit Amsterdam De Boelelaan 1100 1081 HV Amsterdam The Netherlands

## Abstract

Polymer-based nanoparticles (PNPs) find wide application in *e.g.* material and pharmaceutical sciences. In order to determine the quality of produced PNPs and understand their function, analytical characterization is essential. Recently, two-dimensional liquid chromatography (2D-LC) has been introduced as an effective means to probe multiple PNP properties, such as size and composition, in an integrated manner. Such approaches involve transformation of PNPs into their constituents between the two LC dimensions by introducing an organic solvent. Employing light scattering, which is mainly caused by intact PNPs, this paper presents nephelometry as a straightforward technique to assess the percentage of organic solvent needed to achieve full disintegration of PNPs. Empty and dye-loaded poly(lactic-*co*-glycolic acid) (PLGA) based PNPs were exposed to organic solvent-water mixtures of different ratios, while measuring their scattering intensities at a 90° angle when irradiated with a 650 nm light beam. Disruption of the PNPs was marked by a gradual decrease of the scattering intensity with increasing percentages of organic solvent in the PNP suspension. Full disassembly of the PNPs was achieved with 50% acetonitrile in water (v/v). The overall shape of the disassembly profiles and the minimum percentage of organic solvent needed for full PNP disintegration was found to be largely independent of the type and quantity of dye loaded, or the amount of polymer used for PNP formation.

## Abstract 2

Polymer-based nanoparticles (PNPs) find wide application in *e.g.* material and pharmaceutical sciences.

## Graphical abstract

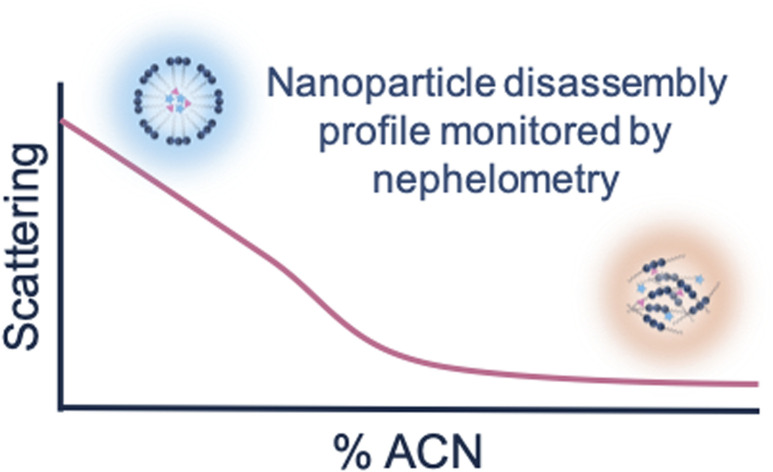

## 1 Introduction

Nanoparticles (NPs) are widely abundant in modern daily life, either as useful materials (*e.g.* coatings and drug-delivery vehicles)^1–3^ or as pollutants of the environment and organisms (*i.e.* nanoplastics).^4^ NPs may be made of metals or metal oxides, but many are comprised of macromolecules, such as lipids or polymers. Polymer NPs (PNPs) can be entirely composed of a polymer, such as polystyrene (PS) or polyurethane (PUR), or consist of a (bio)polymer-based carrier that encapsulates a low-molecular weight compound. The latter class of PNPs are frequently used to achieve controlled and/or delayed release of drugs after administration and studying their disintegration will be the topic of this paper.

Characterization of produced PNPs is important to control and understand their function, efficacy, stability, environmental fate and/or uptake.^5^ The complex and often heterogeneous composition of PNPs generally requires several analytical methods of different selectivity to assess all sample dimensions, such as size, size distribution, polymer composition and overall constitution of PNPs.^6^ Determination of PNP sizes is often achieved by using techniques based on *e.g.* light scattering or electron microscopy. Compositional analysis of PNPs may, for example, include the monitoring of degradation of the polymer molecules that make up biodegradable PNPs used for drug delivery, or the determination of the quantity and stability of PNP-encapsulated species, such as active pharmaceutical ingredients. These determinations commonly involve liquid chromatographic (LC) techniques as size-exclusion chromatography (SEC) or reversed-phase (RP) LC.

Analysis of PNP components is normally carried out after full disruption of the PNP structure into its constituting molecules. Waterborne PNP formulations are commonly synthesized using so-called nanoprecipitation,^7^ in which (partly) hydrophobic polymer molecules are dispersed in an aqueous solvent. Driven by hydrophobic interaction, the polymer molecules will assemble into NPs resulting in a suspension. Disintegration of such PNPs can be achieved quite straightforwardly by adding an excess of a water-miscible organic solvent to the suspension to disrupt the intermolecular bonding and solubilize the individual NP components.^8,9^

As indicated above, separate analytical techniques are needed to assess the various PNP characteristics, which can be tedious and time consuming. Moreover, the individual techniques provide overall sample properties and, for instance, do not allow evaluation of PNP composition as a function of PNP size. This drawback could, at least partly, be circumvented by involving a multi-dimensional analytical approach, probing different sample properties in an integrated fashion. Two-dimensional (2D) LC, in which two chromatographic modes of orthogonal selectivity are combined, has demonstrated to be a very powerful approach to separate and assess different molecular dimensions of a sample simultaneously.^6,10^ Therefore, application of 2D-LC for comprehensive PNP analysis is an attractive option, but also quite challenging, as it requires (i) employment of an LC mode suitable for intact NP analysis, and (ii) an online means to achieve transformation of PNPs into its constituents between the two LC dimensions. Hydrodynamic chromatography (HDC) is currently the only LC technique that can provide size-based separation of PNPs in the range of 10 up to 1000 nm. So far, two online 2D-LC methods suitable for PNP analysis have been reported. Pirok *et al.* designed and applied a comprehensive HDC × SEC system for the analysis of polyacrylate (co-)polymer NPs.^11,12^ Intact PNPs were first separated by size using HDC and subsequently the molecular-weight distribution of the constituting polymers was established by SEC as a function of NP size. Verduin *et al.* presented an online HDC × RPLC platform for the characterization of drug-delivery systems using PNPs with encapsulated hydrophobic dyes as a model species.^13^ The NP payloads could be profiled over the entire NP size distribution in a single run. Both reported 2D-LC methods involved an online NP-disassembly step to disrupt the PNP structure and dissolve its individual constituents. Whereas NP disassembly by addition of an excess organic solvent usually suffices for an offline one-dimensional LC analysis, the choice of the proper composition of the NP-disrupting solvent needs more careful consideration in 2D-LC. Next to the capability to completely disrupt the target NP and dissolve its components, the used solvent should also avoid incompatibility issues with the second-dimension LC mode. Therefore, in order to allow selection of an appropriate solvent for online disassembly of PNPs, a simple method that can monitor PNP transformation in solution would be highly useful.

Dynamic light scattering (DLS) could provide a potential means to monitor presence of NPs, but requires specialized equipment. Transmission electron spectroscopy (TEM) can detect NPs, however, it is costly and requires solvent removal to obtain dry particles and, therefore, is less suited for routine assessment of NPs in solution. The presence of small particles suspended in a solvent can, in principle, also be detected quite straightforwardly with optical techniques such as turbidimetry or nephelometry.^14,15^ Briefly, in turbidimetry, an ultraviolet-visible (UV-Vis) absorbance spectrophotometer is used to measure light transmission of a sample. As NPs cause light scattering, their presence in a probed solution will induce an apparent absorbance, which can be monitored, *e.g.* as function of solvent composition.^16–18^ In nephelometry, a fluorescence spectrophotometer is used to directly measure the light scattering caused by NPs in solution. Nephelometry has been employed as a detection principle for proteins,^19–21^ biomass,^22,23^ and atmospheric particles,^24^ but to the best of our knowledge, not for the monitoring of the disassembly of PNPs. In comparison with turbidimetry, nephelometry is superior in terms of sensitivity, as light scattering is measured at a 90° angle to the incident light with corresponding low background signals.

In the present work, we evaluated if and how a simple nephelometry approach can be used to study PNP integrity as a function of solvent composition. Waterborne PNPs, comprising a triblock copolymer of polyethylene glycol (PEG) and poly(lactic-*co*-glycolic acid) (PLGA), were used as model PNPs. PLGA–PEG–PLGA is a biodegradable copolymer that is frequently used as drug carrier in pharmaceutical formulations. Using a commercial fluorescence spectrometer, we investigated the PLGA-PEG-PLGA-NP scattering intensity as a function of the percentage organic solvent in water and of time. From these data, we established ‘disassembly profiles’ of the PLGA-PEG-PLGA NPs, which indicate at what solvent composition the PNP under study is fully disintegrated. Subsequently, we employed the nephelometry method to assess the disassembly of several PLGA-PEG-PLGA-NP formulations without and with payload dyes of various hydrophobicities.

## 2 Materials and methods

### 2.1 Chemicals and PNP preparations

Acetone (technical grade) and tetrahydrofuran (THF, unstabilized ≥99.5%) were purchased from VWR chemicals (Fontenay-sous-Bois, France). Coumarin-6 (98%), curcumin (from *Curcuma longa*), poly(lactide-*co*-glycolide)-*block*-poly(ethylene glycol)-*block*-poly(lactide-*co*-glycolide) (PLGA–PEG–PLGA, average *M*_n_ 6000–12 000), and Sudan-IV were obtained from Sigma Aldrich (Darmstadt, Germany). Acetonitrile (ACN, LC-grade) was obtained from Biosolve BV (Valkenswaard, the Netherlands). The water used was deionized (Arium 611UV; Sartorius, Germany, resistivity 18.2 MΩ cm).

The PNP test samples were synthesized by quickly dispersing a solution of PLGA–PEG–PLGA in water (nanoprecipitation). For that, a solution of 6 g L^−1^ PLGA–PEG–PLGA copolymer was prepared in acetone. Samples of empty PNPs were prepared by injecting 400 or 200 μL of the copolymer solution in 8 mL water, followed by 15 min of sonication. Subsequently, acetone was evaporated from the aqueous suspensions in an oven at 40 °C for 5 h. For the production of dye-loaded PNPs, 400 μL of copolymer solution was first mixed with 200 μL dye solution, after which the 600 μL was dispersed in 8 mL water. Dye solutions were prepared in acetone at concentrations of 250 and 500 mg L^−1^. The sizes of the produced PNPs were determined by HDC according the procedure described in ref. 13. For that, a calibration curve was constructed using size-certified polystyrene NPs. The mean size of the empty, curcumin-loaded, coumarin-6-loaded and Sudan-IV-loaded PNPs was measured to be 29, 44, 41 and 72 nm, respectively.

### 2.2 PNP disassembly experiments

Produced PNPs were exposed to various percentages of organic solvent (ACN or THF) in water. To 125 μL of PNP suspension in water, 875 μL of an organic solvent–water mixture of different ratios was added, leading to the following overall percentages (v/v) of ACN in the final suspension: 0, 10, 20, 40, 45, 50, 55, 60, and 70% (10% only for the empty PNPs). When using THF, the final suspensions contained the following overall percentages (v/v) of THF: 0, 10, 20, 30, 40, 43, 50, 60, and 70%. Upon addition of the organic solvent–water mixture, samples were immediately homogenized with a vortex mixer and then directly transferred to a cuvette for analysis by nephelometry.

### 2.3 Nephelometry

Nephelometry measurements were conducted on a Varian Cary Eclipse fluorescence spectrophotometer. The instrument is equipped with a xenon flash lamp and a red-extended photomultiplier for detection. The excitation wavelength was 650 nm (power at the sample, 0.3 mW). Sample scattering was recorded 5 times per sample by scanning the emission wavelength from 600 to 700 nm at a rate of 600 nm min^−1^. The spectral bandwidths for excitation and emission were 2.5 and 5.0 nm, respectively. The excitation filter was set at “auto” (thus blocking any interference from 325 nm light) and the emission filter was set to “open”. Factors like detector dark current or fluorescence background are automatically corrected for by scanning over a 100 nm wavelength range. Samples were measured (*n* = 5) in a quartz cuvette, which had an optical path length of 10.0 mm and was closed with a lid.

### 2.4 Data processing

Matlab R2023b was used for handling the recorded scatter data and creating graphs. The trapz function was used to determine the area of the observed scattering peak between 640 and 660 nm. For each PNP experiment, the average areas, standard deviations, and relative standard deviations (RSDs) were calculated for the 5 replicates.

## 3 Results and discussion

### 3.1 Monitoring PNP disassembly by nephelometry

#### 3.1.1 Basic performance

**Fig. 1:**
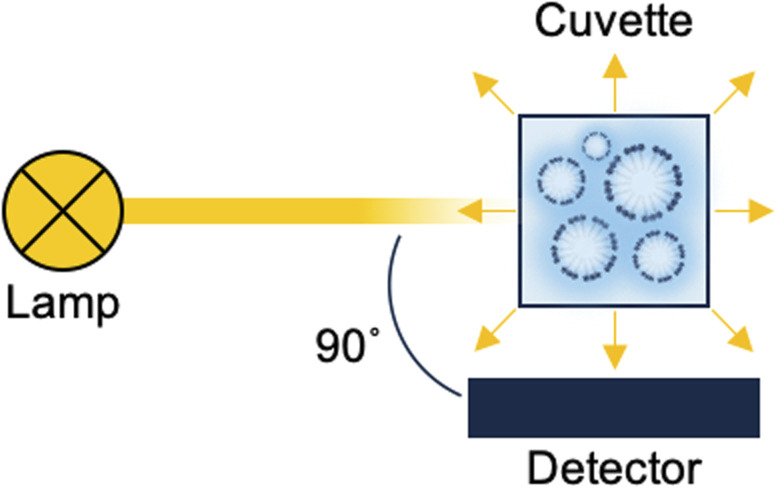
Simplified schematic of the nephelometry set-up. When a light beam irradiates a cuvette filled with a PNP suspension, light is scattered in all directions. The scatter emitted at a 90° angle from the incident beam is detected.

We first checked whether the presence of the PNPs in solution can be measured with our plain nephelometer (Fig. 1). An excitation wavelength of 650 nm was selected in order to avoid absorbance or fluorescence by the encapsulated dyes in some of the PLGA-NP samples that would otherwise affect the measured scattering signal.

**Fig. 2:**
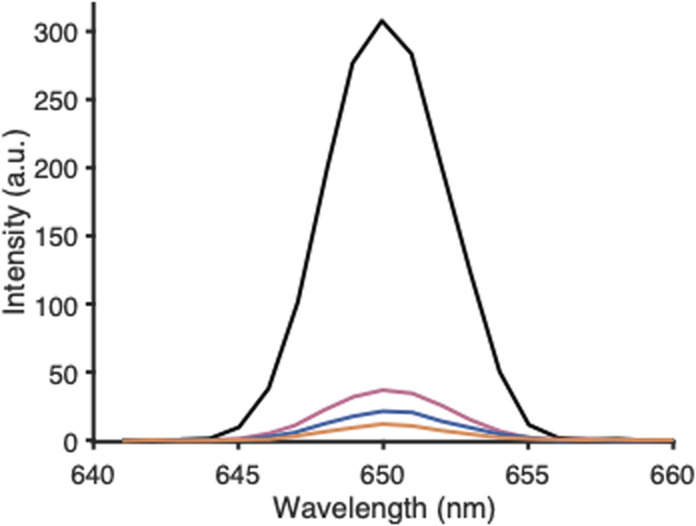
Scattering intensity measured over a narrow wavelength range for empty PLGA-PEG-PLGA NPs in water (black trace), for blank water (blue trace), for blank ACN (orange trace), and for PLGA–PEG–PLGA copolymer dissolved in ACN (150 mg L^−1^) (pink trace). Excitation wavelength, 650 nm; 400 μL of 6 g L^−1^ PLGA–PEG–PLGA copolymer used for PNP preparation.

Fig. 2 depicts the scattering signal as a function of emission wavelength, recorded for (i) a produced suspension of empty PLGA NPs, (ii) pure water, (iii) pure ACN, and (iv) a solution of PLGA–PEG–PLGA polymer in ACN. The polymer concentration in the latter sample equalled the overall polymer concentration in the PLGA NP sample used for (i). The sample with PNPs showed a clearly higher scattering intensity with respect to the reference solvents and polymer solution. The solvent and dissolved polymer molecules cause Rayleigh scattering, but when PNPs in the 10–100 nm range are present in the probed solution, apparently Mie scattering by the NPs becomes the dominant contributor to the overall observed scattering signal. The relative standard deviation (RSD) obtained for five successive scattering measurements of the same sample was generally below 5%, showing satisfactory repeatability of the method. From this experiment, we assumed that the used method, in principle, provides a simple means to monitor PNPs in an aqueous suspension.

#### 3.1.2 Exposure of PNPs to organic solvent

**Fig. 3:**
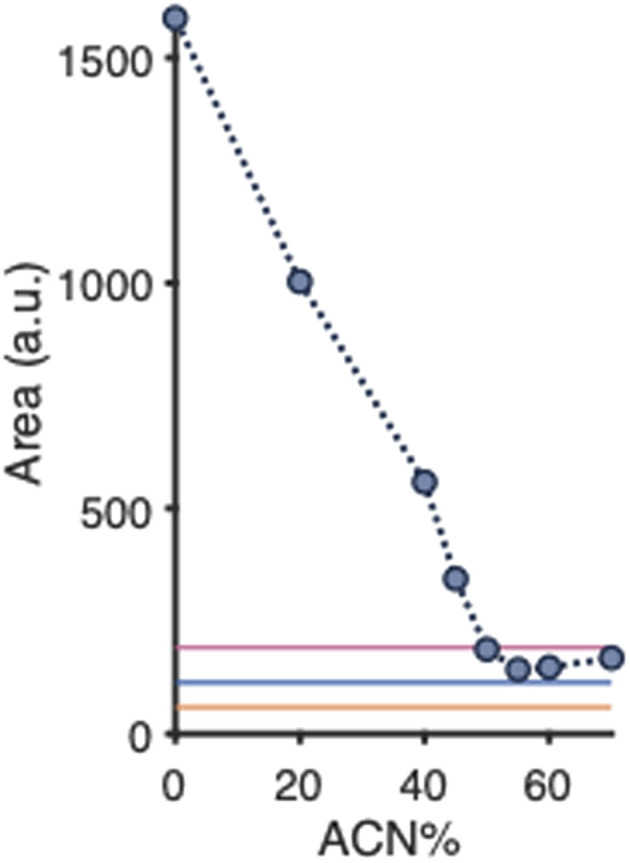
Mean scattering intensities (*n* = 5) of empty PLGA-PEG-PLGA NPs exposed to various percentages of ACN (v/v) in water. Colored lines represent the Rayleigh scattering intensities obtained for PLGA–PEG–PLGA copolymer dissolved in ACN (150 mg L^−1^) (pink), blank water (blue), and blank ACN (orange); 400 μL of 6 g L^−1^ PLGA–PEG–PLGA copolymer was used for PNP preparation. Relative standard deviations are listed in Table S1. Blank signals of various percentages of ACN in water are depicted in Fig. S1.

As outlined in the Introduction, we were particularly interested in the determination of the minimal amount of organic solvent needed to fully disintegrate PNPs into their constituents. Therefore, 125 μL aliquots of the produced PLGA-PEG-PLGA NP suspension were mixed with 875 μL ACN–water of different ratios, yielding overall ACN percentages of 0% to 70% (v/v). Notably, the total concentration of polymer in each mixture was equal. Immediately after mixing, the scattering of the sample was assessed using nephelometry. Fig. 3 depicts the recorded scattering intensity as a function of the percentage of ACN in the sample; blank signals of various percentages of ACN in water are depicted in the SI (Fig. S1). The scattering signal clearly decreases with an increasing percentage of ACN. At about 50% ACN the scattering intensity is similar to the scattering observed for blank solvents (as also depicted in Fig. 3), and stays around that level for further increasing ACN percentages. We assume that ACN affects the PNP integrity and reduces the number and/or size of the PLGA-PEG-PLGA NPs. At 50% ACN and higher, PNP scattering is no longer observed, *i.e.*, the PNPs most probably are entirely disrupted. From this experiment we concluded that the simple nephelometry approach can be used to study the disassembly of PNPs.

**Fig. 4:**
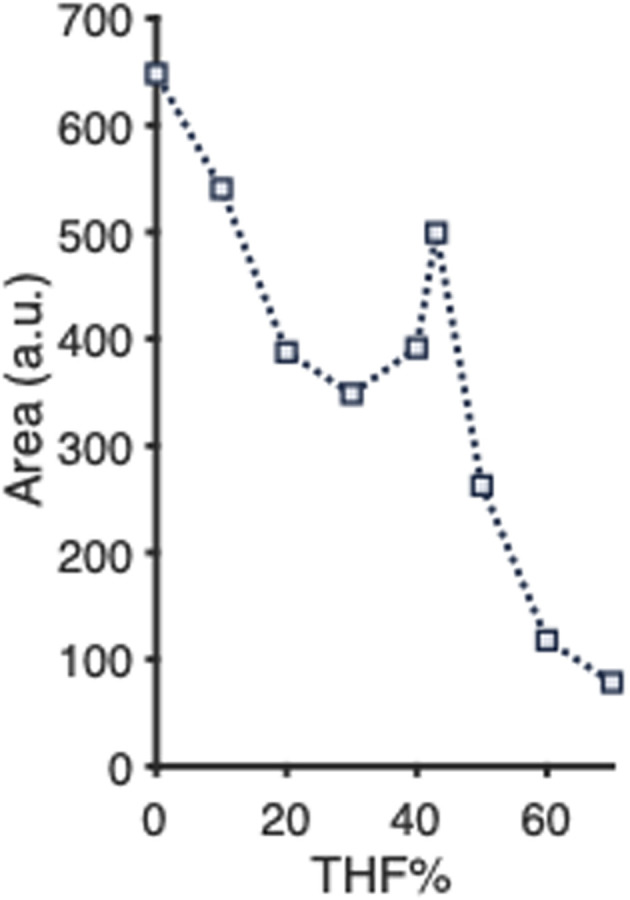
Mean scattering intensities (*n* = 5) of empty PLGA-PEG-PLGA NPs exposed to various percentages of THF (v/v) in water. 200 μL of 6 g L^−1^ PLGA–PEG–PLGA copolymer was used for PNP preparation. Relative standard deviations are listed in Table S2.

We also exposed another batch of empty PLGA-PEG-PLGA NPs to THF, following the same procedure as described above for ACN. Addition of THF to the PNP suspension also causes a decrease of the scatter signal, indicating disruption of the PNPs (Fig. 4). Full disintegration seems to be attained at about 60% (v/v) of THF in water. However, in contrast to ACN, the disassembly profile does not follow a gradual decrease of the scattering signal with increasing THF percentage. A local increase of scattering is observed around 43% THF. We do not know the reason for this observation, but the phenomenon was also observed when the experiment was repeated on both the same and a freshly prepared batch of PLGA-PEG-PLGA NPs. As the percentage of organic solvent that is needed for full disassembly was somewhat lower for ACN (50%) than for THF (60%), ACN was used in further experiments.

Scatter intensities for the studied NP samples were more than sufficient for a reliable nephelometric measurement. For much lower NP levels, the detection will be limited by the shot noise on the solvent Rayleigh scattering intensity. In that case, the signal-to-noise ratio of the measurement could still be improved by working with wider spectral bandwidths or slower wavelength scanning.

#### 3.1.3 Stability of exposed PNPs in time

**Fig. 5:**
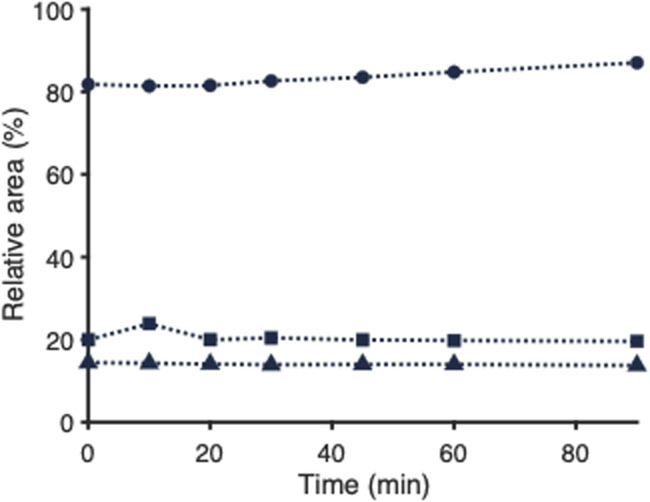
Mean relative scattering intensities (*n* = 5) monitored over time of empty PLGA-PEG-PLGA NPs exposed to 10% (circles), 45% (squares), and 70% (triangles) of ACN in water (v/v). Values (%) are relative to the scattering intensity of empty PLGA-PEG-PLGA NP suspended in pure water (no ACN added). Relative standard deviations are listed in Table S3.

For the correct interpretation of a PNP ‘disassembly profile’ as depicted in Fig. 3, it is important to know whether the PNP disassembly occurs instantaneously when exposed to ACN and whether the resulting PNP status is stable or that disintegration evolves further in time. Immediate disruption is also critical for online PNP transformation in a 2D-LC set-up. In that case, PNP disassembly should happen fast to ensure that the PNP constituents are introduced correctly into the second LC dimension for further analysis. Therefore, the scattering intensity of empty PLGA-PEG-PLGA NPs exposed to ACN was monitored over a period of 90 min. Fig. 5 shows the results obtained for PNP exposure to 0%, 10%, 45% and 70% of can, measured at regular time intervals. Interestingly, at each ACN percentage, the scatter intensity is quite constant in time, that is, after a quick initial drop in scattering when the PNPs are exposed to ACN, no further decline of the scattering signal occurs over time. From this, we infer that a certain part of the PNP population disintegrates promptly at a specific percentage ACN. Whether a certain fraction of PNPs consists of stable particles or whether a dynamic equilibrium is reached cannot be determined with this data.

### 3.2 Nephelometric assessment of the disassembly of PLGA-NP formulations

#### 3.2.1 Polymer amount used in NP preparation

**Fig. 6:**
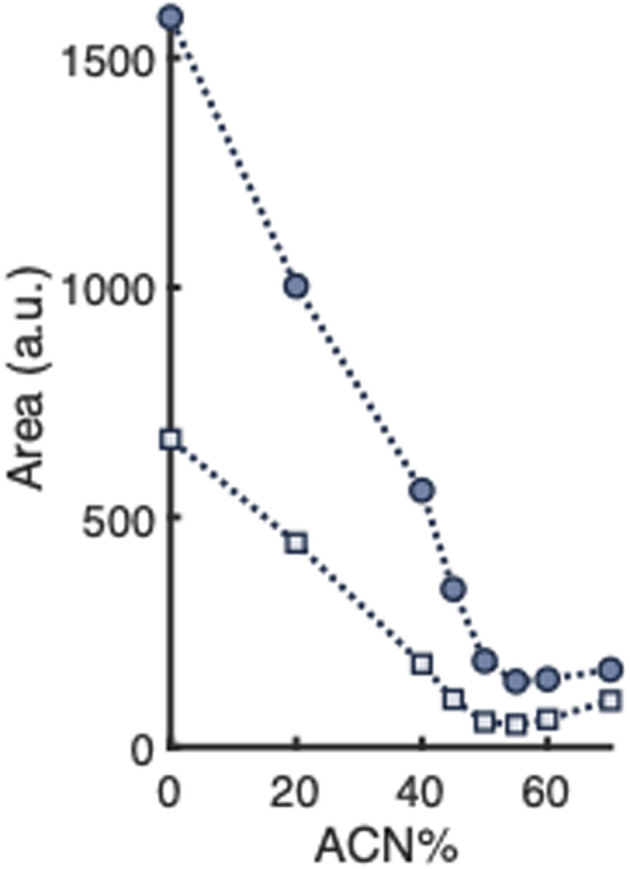
Mean scattering intensities (*n* = 5) of empty PLGA-PEG-PLGA NP formulations prepared using high (dark blue circles) or low (light blue squares) amounts of copolymer and exposed to various percentages of ACN (v/v) in water. Relative standard deviations are listed in Table S1.

For the preparation of PNPs by nanoprecipitation, different amounts of polymer can be used, which might affect the percentage organic solvent needed to disintegrate the PNPs. In order to study this, two formulations of empty PNPs were prepared by separately dispersing 200 or 400 μL PLGA–PEG–PLGA solution (6 g L^−1^) in 8 mL water. Fig. 6 shows the disassembly profiles of the resulting PNP samples when exposed to ACN and analyzed by nephelometry. The scattering intensity at 0% ACN is lower when less polymer is dispersed, most probably because fewer PNPs are formed. However, the shapes of the disassembly profiles of the PNP samples are very similar, reaching the minimum scattering signal at about 50% ACN. It seems that the amount of polymer used for the PNP formulation does not affect the ACN percentage at which the studied PNPs are fully disintegrated.

#### 3.2.2 PNPs with encapsulated dyes

**Fig. 7:**
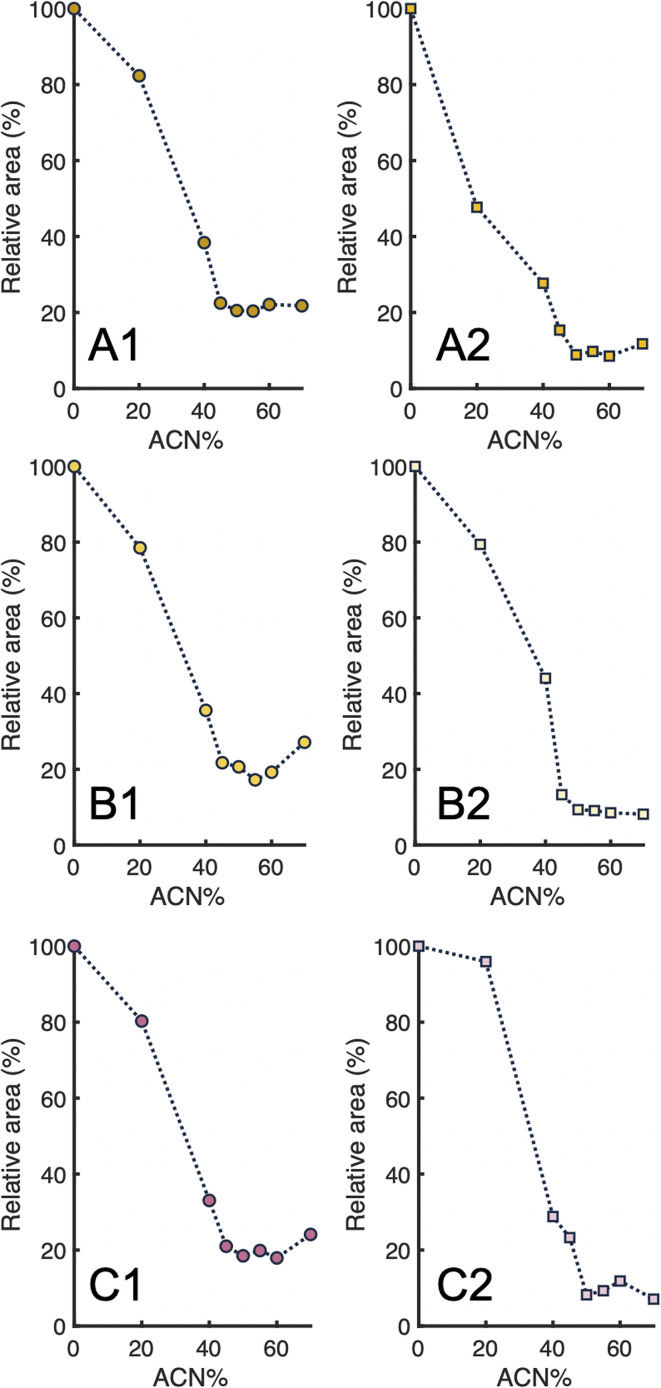
Mean relative scattering intensities (*n* = 5) of dye-loaded PLGA-PEG-PLGA NP formulations prepared using high (left, annotated with 1) and low (right, annotated with 2) amounts of dye and exposed to various percentages of ACN (v/v) in water. Loaded dye: (A) curcumin; (B) coumarin-6; (C) Sudan-IV. Values (%) are relative to the scattering intensity of the PLGA-PEG-PLGA NP suspended in pure water (no ACN added). Relative standard deviations are listed in Table S1.

PLGA-PEG-PLGA NPs find widespread application as carriers in drug-delivery systems (DDS). During preparation of these formulations, an active pharmaceutical ingredient is encapsulated into the NPs. 2D-LC analysis of such DDS would require online disruption of the NPs by an organic solvent. To mimic DDS, PLGA-PEG-PLGA NPs were prepared with three different encapsulated dyes of increasing hydrophobicities: curcumin, coumarin-6, and Sudan-IV. For each dye, PNP samples with two different dye loads (low and high) were prepared. We used the nephelometry setup to study the disintegration of the dye-encapsulated PNPs as a function of the percentage of ACN in the solvent. The resulting disassembly profiles are plotted in Fig. 7. Overall, the dye-loaded PNPs show quite similar disassembly behavior when exposed to ACN, regardless the type and load of dye. The PNPs with higher dye load show somewhat higher scattering signals, but the scattering gradually decreases when the percentage of ACN is increased, indicating disintegration of part of the NPs. For all dye-loaded PNP samples, minimum scattering intensity is reached at about 50% ACN, corresponding to complete NP disruption. Apparently, the amount and type of encapsulated compounds do not affect the specific ACN percentage at which the PLGA-PEG-PLGA PNPs fully disintegrate. This implies that one fixed percentage of organic solvent can be selected for online disassembly, which is an evident advantage when using 2D-LC for this type of PNPs.

## 4 Conclusions

This work describes how a straightforward nephelometry approach with a commercial spectrofluorometer can be used as a tool to study PNP disassembly in solution. Such information is, for example, highly useful when PNP transformation has to be achieved between two LC modes in a 2D-LC setup. Empty and dye-loaded PLGA-PEG-PLGA NPs, employed here to mimic DDS NPs, were exposed to different amounts of ACN in the diluent and probed by nephelometry. The steady decrease of scattering intensity with increasing ACN percentage indicated the gradual disintegration of the PNPs. The (partial) disassembly appeared to happen within seconds when exposed to ACN, which shows promise for achieving quick PNP transformation in online systems. The amount of NP-constituting polymer nor the amount of encapsulated dye appeared to affect the ACN percentage needed for full NP disintegration, which was about 50% of ACN in water for the studied PLGA-PEG-PLGA NPs. We think that the nephelometry approach shows good potential for investigating the disruption of other types of NPs, such as the disassembly of oligonucleotide-loaded lipid NPs using neutral surfactants. Moreover, the nephelometric method could be used to monitor the stability and disintegration of NPs used for drug delivery in real time.

## Author contributions

J. V.: conceptualization, methodology, formal analysis, investigation, data curation, visualization, writing – original draft; N. S.: investigation; F. A.: conceptualization, methodology, review & editing; G. S.: conceptualization, supervision, funding acquisition, project administration, writing, review & editing.

## Conflicts of interest

There are no conflicts to declare.

## Supplementary Material

AY-017-D5AY00659G-s001

## Data Availability

The nephelometry data presented in this article are available at the following zenodo repository link: https://doi.org/10.5281/zenodo.15195542. Supplementary information is available. See DOI: https://doi.org/10.1039/d5ay00659g.
