# Supplementary material for: Nephelometry as a simple tool to monitor the disassembly of polymeric nanoparticles
Source: Anal Methods. 2025 Aug 26;17(36):7295–300. doi: 10.1039/d5ay00659g (PMC12405835; doi:10.1039/d5ay00659g)
Supplement: AY-017-D5AY00659G-s001 [file AY-017-D5AY00659G-s001.pdf]

Supplementary Information (SI) for Analytical Methods.
This journal is © The Royal Society of Chemistry 2025

# Supporting Information

# Nephelometry as a simple tool to monitor the disassembly of polymeric nanoparticles

Joshka Verduin[1,2], Niki Simaiaki[1,2], Freek Ariese[3], Govert W. Somsen[1,2*]
[1] Vrije Universiteit Amsterdam, Department of Chemistry and Pharmaceutical Sciences, Amsterdam Institute of Molecular and Life Sciences (AIMMS), Division of BioAnalytical Chemistry, De Boelelaan 1085, 1081 HV Amsterdam, the Netherlands
[2] Centre of Analytical Sciences Amsterdam (CASA), Science Park 904, 1098 XH Amsterdam, the Netherlands
[3] LaserLaB Amsterdam, Department of Physics and Astronomy, Vrije Universiteit Amsterdam, De Boelelaan 1085, 1081 HV Amsterdam, the Netherlands

## Table of Contents

***SI-I Scatter intensities of blank acetonitrile-water*** ........ 2

***SI-II Repeatability of nephelometry measurements*** ........ 3

## SI-I Scatter intensities of blank acetonitrile-water

***Figure S1***. *Mean Rayleigh scattering intensities (n =5) obtained for various percentages of ACN (v/v) in water. For convenient comparison, the scaling of the axes is identical to Figure 3 in the manuscript.*

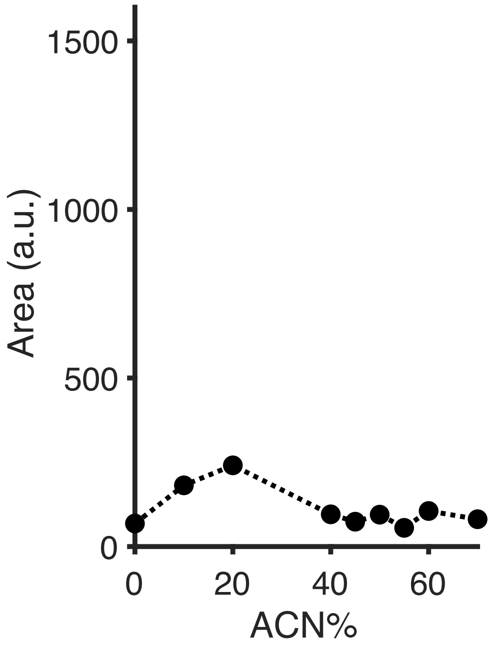

## SI-II Repeatability of nephelometry measurements

***Table S1***. *Relative standard deviations (%) of mean scattering intensities (n=5) of empty and dye-loaded NPs measured at different ACN percentages in water. Mean intensities are depicted in the indicated figures in the manuscript.*

| | **Empty NPs** (Figures 3 and 6) | | **Curcumin NPs** (Figures 7A1-2) | | **Coumarin-6 NPs** (Figures 7B1-2) | | **Sudan-IV NPs** (Figures 7C1-2) | |
|---|---|---|---|---|---|---|---|---|
| *Polymer/dye amount* | *Low* | *High* | *Low* | *High* | *Low* | *High* | *Low* | *High* |
| **ACN (%)** | | | | | | | | |
| 0 | 0.5 | 0.7 | 3.2 | 0.4 | 4.7 | 9.2 | 1.1 | 1.1 |
| 20 | 2.5 | 2.6 | 1.9 | 4.3 | 19.7 | 8.5 | 6.0 | 0.5 |
| 40 | 2.4 | 1.8 | 9.3 | 0.6 | 5.0 | 5.4 | 3.6 | 3.1 |
| 45 | 2.7 | 10.5 | 1.8 | 7.4 | 16.2 | 16.6 | 15.2 | 1.5 |
| 50 | 3.9 | 8.8 | 7.4 | 1.5 | 2.7 | 12.6 | 2.1 | 1.0 |
| 55 | 3.9 | 2.0 | 6.5 | 5.0 | 8.0 | 1.7 | 13.1 | 2.2 |
| 60 | 6.9 | 1.2 | 3.0 | 3.4 | 4.7 | 6.1 | 4.7 | 1.2 |
| 70 | 4.2 | 2.0 | 28.3 | 1.8 | 9.6 | 17.2 | 3.1 | 5.3 |

***Table S2***. *Relative standard deviations (%) of mean scattering intensities (n=5) of empty NPs measured at different THF percentages in water. Mean intensities are depicted in Figure 4 of the manuscript.*

| **THF (%)** | **RSD (%)** |
|---|---|
| 0 | 0.6 |
| 10 | 1.7 |
| 20 | 0.8 |
| 30 | 2.0 |
| 40 | 6.4 |
| 43 | 5.0 |
| 50 | 1.5 |
| 60 | 4.2 |
| 70 | 5.8 |

***Table S3***. *Relative standard deviations (%) of mean scattering intensities (n=5) of empty NPs exposed to 10%, 45%, and 70% ACN in water for 0-90 min. Mean intensities are depicted in Figure 5 of the manuscript.*

| **Time (min)** | **10% ACN** | **45% ACN** | **70% ACN** |
|---|---|---|---|
| 0 | 1.3 | 0.7 | 3.2 |
| 10 | 1.1 | 1.4 | 3.3 |
| 20 | 0.7 | 1.3 | 3.2 |
| 30 | 0.9 | 2.1 | 2.3 |
| 45 | 1.3 | 0.7 | 1.5 |
| 60 | 0.4 | 1.3 | 2.2 |
| 90 | 0.6 | 1.6 | 2.0 |
